# Trifolin inhibits the calcium-driven contraction pathway in vascular smooth muscle

**DOI:** 10.3389/fphar.2025.1573483

**Published:** 2025-05-30

**Authors:** Renfeng Li, Jinkong Wu, Meizhu Wu, Farman Ali, Yanyan Yang, Hong Chen, Zhi Guo, Dawei Lian, Aling Shen, Jun Peng

**Affiliations:** ^1^ Academy of Integrative Medicine, Fujian University of Traditional Chinese Medicine, Fuzhou, Fujian, China; ^2^ College of Integrative Medicine, Fujian University of Traditional Chinese Medicine, Fuzhou, Fujian, China; ^3^ Fujian Key Laboratory of Integrative Medicine on Geriatrics, Fujian University of Traditional Chinese Medicine, Fuzhou, Fujian, China; ^4^ Fujian Collaborative Innovation Center for Integrative Medicine in Prevention and Treatment of Major Chronic Cardiovascular Diseases, Fuzhou, Fujian, China; ^5^ Innovation and Transformation Center, Fujian University of Traditional Chinese Medicine, Fuzhou, Fujian, China

**Keywords:** trifolin, hypertension, vasoconstriction, calcium signaling pathway, smooth vascular muscle

## Abstract

Trifolin, a bioactive component of the Qingda granule, has demonstrated significant antihypertensive potential; however, its precise mechanisms of action remain largely unknown. This study aimed to investigate the antihypertensive effects of trifolin and unravel its underlying molecular mechanisms. The influence of trifolin on vascular contraction and relaxation and its regulatory effects on ion channels were evaluated through a vascular tension experiment. Morphological changes in the aortic tissues of mice with angiotensin Ⅱ-induced hypertension and the expression profiles of contraction-associated proteins were analyzed via hematoxylin-eosin staining and immunohistochemistry. Additionally, trifolin’s impact on calcium ion dynamics and contraction-associated protein expression in angiotensin Ⅱ-activated vascular smooth muscle cells (VSMCs) was determined through calcium flux assays and western blot analyses. Trifolin treatment decreased the constriction of isolated abdominal aortic rings induced by norepinephrine, KCl, and angiotensin Ⅱ in an endothelium-independent manner and extracellular Ca^2+^ influx induced by these three substances and thapsigargin. Moreover, trifolin treatment significantly reduced the abdominal aortic wall thickness and downregulated the expression of store-operated channels channel proteins (STIM1 and ORAI1) and calcium signaling-related proteins (CaM, myosin light chain kinase, and p-MLC2) in the abdominal aorta of hypertensive mice and angiotensin Ⅱ-induced VSMCs. In conclusion, calcium signaling inhibition may underlie trifolin’s antihypertensive effects and its ability to ameliorate vascular function. These findings offer new therapeutic insights for hypertension treatment.

## Graphical Abstract

**Figure:**
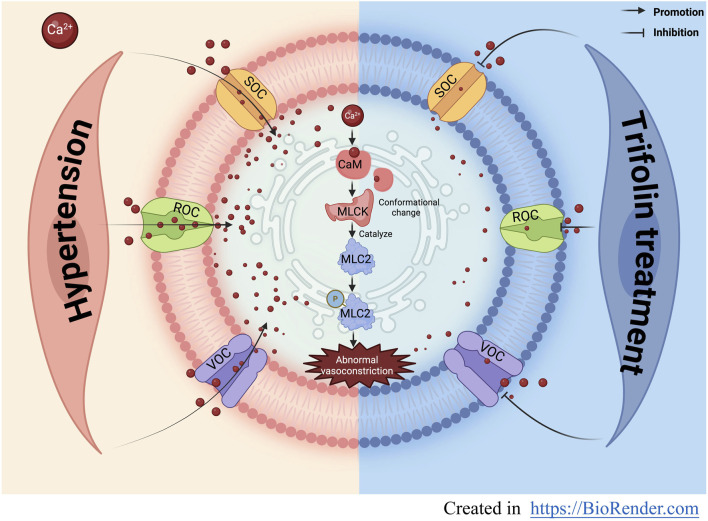
Trifolin may exert antihypertensive effects and ameliorate vascular function by inhibiting the influx of extracellular calcium mediated by calcium ion channels .

## 1 Introduction

Hypertension, a chronic cardiovascular disorder, continues to be highly prevalent and has a strong link to increased morbidity and mortality, thereby remaining to be a global health challenge (Labarthe and Dunbar, 2012; Zhang et al., 2023). Its pathogenesis is primarily driven by elevated vascular resistance, stemming from structural and functional changes including endothelial dysfunction, vascular smooth muscle hypercontractility, and arterial remodeling (Savoia et al., 2011; Montezano et al., 2015; Touyz et al., 2018). These pathological alterations contribute to disease progression and complications; thus, more precise and targeted therapeutic strategies are needed. Among the numerous mechanisms regulating vascular function, calcium signaling plays a pivotal role, given that increased intracellular Ca^2+^ concentrations in vascular smooth muscle cells (VSMCs) induce vasoconstriction (Allen and Walsh, 1994; Matsuda, 1997; Ikebe, 2008; Goulopoulou and Webb, 2014). Therefore, calcium channel inhibition may be a key strategy in antihypertensive therapy.

On the basis of the pivotal role of calcium signaling in VSMC contraction and blood pressure regulation, calcium influx is primarily mediated by three types of calcium channels: voltage-dependent channels (VDCs), receptor-operated channels (ROCs), and store-operated channels (SOCs) (Matsuda, 1997; Chin and Chueh, 2000; Wynne et al., 2009; Misárková et al., 2016). These channels collectively regulate intracellular Ca^2+^ concentrations, initiating a cascade that induces conformational changes in calmodulin (CaM) and activates myosin light chain kinase (MLCK) (Fang et al., 2023). Subsequently, MLCK activation phosphorylates the myosin light chain, causing vascular constriction (Allen and Walsh, 1994; Ikebe, 2008; Walsh, 2011; Hill and Meininger, 2016). Although calcium channel-targeting drugs are widely used in clinical practice, their therapeutic efficacy is often constrained by issues such as drug resistance and inadequate therapeutic responses (Reinhart et al., 2023), likely attributable to the complex regulatory mechanisms of calcium channels and their diverse physiological roles in vascular homeostasis (Thorneloe and Nelson, 2005; Wynne et al., 2009; Martinsen et al., 2014; Touyz et al., 2018). Therefore, exploring the calcium signaling pathways further and developing innovative, targeted therapeutics are crucial to address these limitations and improve the hypertension therapy.

Qingda granules (QDG) exhibited a significant antihypertensive effect with low-to-moderate risk profiles in clinical trials (Qu et al., 2024). They were also effective in reducing blood pressure in both rats with spontaneous hypertension and mice with angiotensin Ⅱ (Ang Ⅱ)-induced hypertension via enhanced vasodilation (Xiao et al., 2016; Liu et al., 2017; Hei et al., 2020; Wu et al., 2020; Zhang et al., 2020; Cheng et al., 2021; Long et al., 2021; Long et al.,2024; Chen et al., 2022;Reinhart et al., 2023). Among its active components, trifolin, a flavonoid compound, demonstrated promising hypotensive effects in preliminary experiments. However, the precise mechanisms underlying calcium signaling modulation and vascular function improvement by trifolin remain inadequately understood, necessitating further investigation.

**FIGURE 1:**
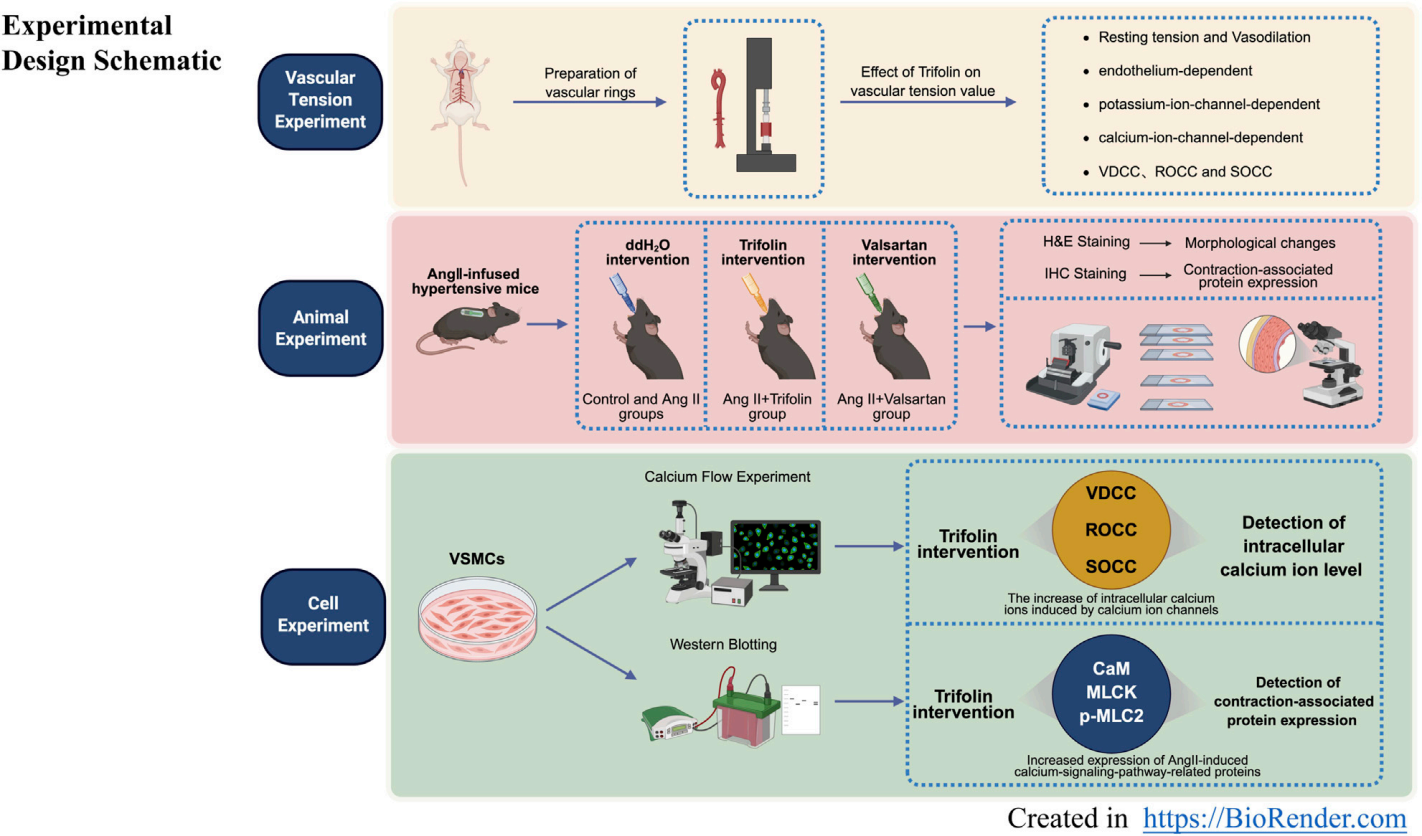
Experimental design schematic. Experimental design schematic for *ex vivo*, *in vivo*, and *in vitro* experiments.

Hence, this study aimed to elucidate the molecular mechanisms by which trifolin exerts its antihypertensive effects, focusing on its regulation of calcium signaling pathways (Figure 1). By investigating these pathways, we could provide innovative insights into trifolin’s therapeutic potential and strengthen scientific evidence for its application in hypertension treatment.

## 2 Materials and methods

### 2.1 Reagents

Trifolin (purity >98%) was obtained from Chengdu Alfa Biotechnology Co., Ltd. (Chengdu, Sichuan, China). Other chemicals such as KCl, valsartan (Val), norepinephrine bitartrate (NE), acetylcholine chloride (ACh), tetraethylammonium chloride (TEA), 4-aminopyridine (4-AP), Nω-Nitro-L-arginine methyl ester hydrochloride (L-NAME), indomethacin (Indo) and verapamil (Ver) were purchased from Sigma-Aldrich (St. Louis, MO, United States). Glibenclamide (Gli) and thapsigargin (TG) were obtained from MedChemExpress (Shanghai, China). Calcium chloride solution (CaCl_2_) and barium chloride dihydrate (BaCl_2_) were purchased from Macklin (Shanghai, China). Ang Ⅱ was ordered from Abcam (Cambridge Science Park, Cambridge, United Kingdom). Fluo-4/AM and fetal bovine serum (FBS) were purchased from Thermo Fisher Scientific (Waltham, MA, United States). Antibodies against MLCK (Cat. No. 48846), p-MLC2 (Cat. No.11114), and GAPDH (Cat. No. 52902) were obtained from Signalway Antibody (College Park, MD, United States). Antibodies against STIM1 (Cat. No. 1156-1-AP) and ORAI1 (Cat. No. 28411-1-AP) were purchased from ProteinTech (Rosemont, IL, United States). Antibody against CaM (Cat. No. 4830s) was purchased from Cell Signaling Technology (Danvers, MA, United States). Antibody against MLC2 (Cat. No. AB92721) was obtained from Abcam (Cambridge Science Park, Cambridge, United Kingdom). UltraSensitive™ SP (Mouse/Rabbit) IHC kit (Cat. No. KIT-9720, including secondary antibodies) was obtained from Maixin Biotechnology (Shanghai, China). Hematoxylin-eosin (H&E) kit (Cat. No. G1100-500) was purchased from Solarbio Technology (Beijing, China). Flunarizine dihydrochloride and YM-58483 were purchased from MedChemExpress (Shanghai, China). Alanine aminotransferase (ALT), aspartate aminotransferase (AST), blood urea nitrogen (BUN), and creatinine (CRE) assay kits were obtained from Nanjing Jiancheng Bioengineering Institute (Nanjing, China).

### 2.2 Animals

To verify the effects of trifolin on the morphological change and contractile function of the abdominal aorta in hypertensive mice, we conducted an experiment using 36 male C57BL/6 mice (8 weeks old), which were purchased from Hangzhou Medical College (Certificate ID: SCXK 2019-0002; Hangzhou, China) and housed under specific pathogen-free conditions at the Animal Center of Fujian University of Traditional Chinese Medicine. The Animal Care and Use Committee of Fujian University of Traditional Chinese Medicine approved our study protocol (Approval No. 2022176). These mice were randomly divided into six groups: control group, Ang Ⅱ group, valsartan-treated group (10 mg/kg/day), and trifolin-treated groups (0.1, 1, and 10 mg/kg/day). Treatments were administered intragastrically once daily for 4 weeks.

To determine the effect of trifolin on the abdominal aorta’s tension value, we used 50 5-week-old Wistar males purchased from Shanghai Slac Laboratory Animal (Certificate ID: SCXK 2022–0004; Shanghai, China). These mice were maintained under specific pathogen-free conditions with a constant temperature of 24°C ± 2°C, a relative humidity of 50%–60%, and a light-dark cycle of 12 h. The animals were provided with water and food throughout the experiment. The committee approved the study protocol (Approval No. 2023039).

### 2.3 Animal model establishment and evaluation

Hypertension was induced in 8-week-old male C57BL/6 mice via continuous subcutaneous infusion of angiotensin Ⅱ (Ang Ⅱ, 500 ng/kg/min) using Alzet 2004 osmotic mini-pumps. Ang Ⅱ was dissolved in physiological saline and loaded into the pump. Control mice received pumps containing saline only. The pump was implanted subcutaneously on the mouse’s back under isoflurane anesthesia. The mouse model in this study meets the criteria of the hypertension model, that is, the systolic blood pressure (SBP) ≥ 140 mmHg, and the diastolic blood pressure (DBP) and the mean arterial pressure (MAP) also increase accordingly (Unger et al., 2020).

### 2.4 Vascular ring experiments

Vascular reactivity was assessed in the isolated abdominal aortic rings. Rats were placed in a closed induction box and supplied with air containing 3%–5% isoflurane. Approximately 1–2 min, the rats could enter the anesthetized state. Subsequently, we rapidly removed and dissected the aorta while avoiding blood vessel damage. All surrounding connective tissues were then cleaned off without damaging the intimal surface of the aorta. Next, the aorta was sliced into 3–4 mm-long rings. These rings were subsequently mounted in a myograph system (Danish Myo Technology, Aarhus, Denmark) containing oxygenated physiological salt solution (PSS) maintained at 37°C and equilibrated in the organ bath for approximately 45 min before the experiment began. During this period, we changed the PSS solution every 15 min to maintain the optimal conditions. Thereafter, we precontracted the aortic rings with KCl or norepinephrine (NE) and added cumulative doses of trifolin to evaluate vasorelaxation.

### 2.5 Calcium flow experiments

A7R5 cells were seeded in confocal dishes at 0.8 × 10^4^ cells/mL, cultivated for 24 h, and then pretreated with or without trifolin (200 μM) for 48 h. At the end of the treatment, fluo-4/AM (5 μM) in Ca^2+^-free HEPES buffered salt solution (HBSS) was added to the dish, followed by incubation for 15 min at 37°C in the dark. Then, we carefully removed the supernatant and washed the cells thrice with Ca^2+^-free HBSS. After incubation with Ca^2+^-containing HBSS for 10 min, the fluorescence intensity and the images were detected using the Volocity software (PerkinElmer, Waltham, MA, United States), and 60 mM of KCl was added during detection. To assess the effect of trifolin on calcium influx and release, we stabilized A7R5 cells with or without trifolin treatment in a Ca^2+^-free HBSS for 10 min after preincubation with flfluo-4/AM (5 μM) and then added CaCl_2_ (2.5 mM), followed by NE, Ang Ⅱ, or thapsigargin stimulation.

### 2.6 Hematoxylin-eosin (H&E) staining

Murine abdominal aorta and heart tissues were dehydrated and embedded in paraffin. These tissues were sectioned at 4 μm thick, deparaffinized, and subjected to H&E staining. Nuclei were stained with hematoxylin for 1 min and the cytoplasm with eosin for 3 s. Stained sections were examined under a light microscope (Leica, Wetzlar, Germany) at ×400 magnification. The average values of media thickness (MT) and lumen diameter (LD) in the proximal and distal regions of blood vessels were precisely measured using ImageJ. Ultimately, the change in blood vessel thickness was evaluated by calculating the MT/LD ratio (Xiao et al., 2016).

### 2.7 Immunohistochemical (IHC) staining

Vascular paraffin sections (4 μm thick) underwent deparaffinization and heat-induced antigen retrieval, followed by incubation with an endogenous peroxidase blocker and a nonspecific staining blocker. Thereafter, the sections were incubated at 4°C with primary antibodies such as MLCK (1:500), CaM (1:50), MLC2 (1:200), p-MLC2 (1:400), STIM1 (1:100), and ORAI1 (1:100) for 12–14 h. Subsequently, we applied biotinylated anti-mouse/rabbit IgG polymer and streptavidin-horseradish peroxidase (HRP). Diaminobenzidine served as a chromogenic agent, and the sections were counterstained with hematoxylin. Finally, we randomly selected three fields of view per section under a 400× light microscope and quantified the positive expression rates by using the ImageJ software (NIH, Bethesda, MD, United States).

### 2.8 Cell culture and treatment

The rat aortic smooth muscle cell line A7R5 was obtained from Wuhan Pricella Biotechnology Co., Ltd. (Wuhan, Hubei, China) and maintained in Dulbecco’s Modified Eagle Medium (DMEM) supplemented with 10% fetal bovine serum and 1% penicillin/streptomycin in a humidified atmosphere with 5% CO_2_ at 37°C. Once the cells reached 80% confluence, they were subcultured. For the experiments, cells were seeded in six-well plates at 0.8 × 10^5^ cells per well. We then cultured the cells in a serum-free DMEM medium for 6 h before adding 1 μM of Ang Ⅱ for hypertension stimulation as a model and being treated with different trifolin concentrations (50, 100, and 200 µM) or equivalent amounts of DMSO as a control. After 48 h of treatment, the cells underwent western blotting, and the calcium flow assays were harvested for further analysis.

### 2.9 Western blotting

After extracting the total proteins from A7R5 cells, we determined the protein concentration by using the bicinchoninic acid assay. Equal amounts of protein were separated via sodium dodecyl sulfate polyacrylamide gel electrophoresis and transferred onto the polyvinylidene fluoride membranes. The membranes were incubated with primary antibodies against MLCK (1:1,000), CaM (1:500), MLC2 (1:1,000), p-MLC2 (1:1,000), STIM1 (1:1,000), ORAI1 (1:1,000), and GAPDH (1:5,000) overnight at 4°C. After incubation with HRP-conjugated secondary antibodies for 2 h, proteins were detected using enhanced chemiluminescence and analyzed using the ImageJ software.

### 2.10 Assay kit experiments

The fresh blood of mice was centrifuged at 3,500 rpm for 10 min. Extract the serum and keep it in the refrigerator at 80°C. Determination of ALT, AST, BUN, and CRE levels by assay kits.

### 2.11 Quantification and statistical analysis

Quantitative data were collected from at least three independent experiments and all statistical analyses were performed using the SPSS statistical program (SPSS/PC+, version 22.0, Chicago, IL, United States). Data were expressed as mean ± SD. Statistical significance was determined using one-way ANOVA followed by Bonferroni’s post hoc test for multiple comparisons. Repeated measures invoked repeated-measures ANOVA. A *p*-value <0.05 was considered statistically significant.

## 3 Results

### 3.1 Trifolin promotes vasorelaxation in the abdominal aortic rings

**FIGURE 2:**
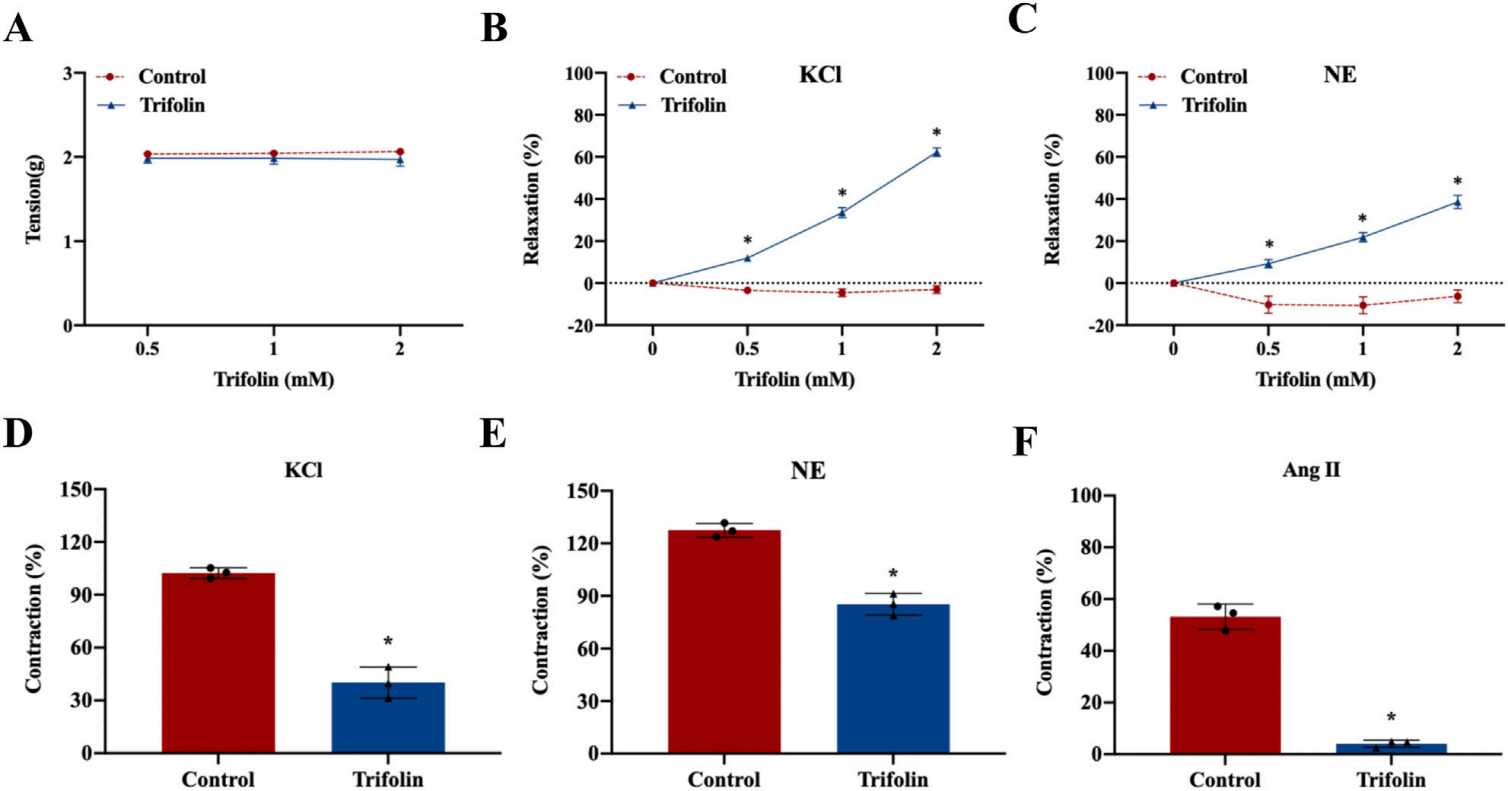
Trifolin promoted vasorelaxation in the abdominal aortic rings. Endothelium-intact aortic rings from Wistar rats were treated with various concentrations of trifolin (0.5, 1, or 2 mM). **(A)** Detection of the resting tension of endothelium-intact aortic rings. **(B–C)** Vasorelaxation of endothelium-intact aortic rings precontracted with KCl (60 mM) **(B)** or NE **(C)**. **(D–F)** Endothelium-intact aortic rings from Wistar rats were pretreated with trifolin (2 mM). Detection of the vasoconstriction of endothelium-intact aortic rings precontracted with KCl **(D)**, NE **(E)**, or Ang Ⅱ **(F)**. Data were shown as the mean ± SD. *p < 0.05 vs. Control group.

To investigate the vasorelaxant effect of trifolin on the abdominal aortic rings in Wistar rats, we initially measured the resting tension of the isolated rings pretreated with various trifolin concentrations (0.5, 1, and 2 mM). Trifolin treatment did not significantly affect the resting tension of the aortic rings (Figure 2A). However, it significantly enhanced the vasorelaxation of endothelium-intact aortic rings precontracted with KCl (60 mM; Figure 2B) or NE (1 μM; Figure 2C). Moreover, trifolin (2 mM) pretreatment significantly decreased the vasoconstriction of endothelium-intact aortic rings stimulated by KCl (60 mM; Figure 2D), NE (1 μM; Figure 2E), or Ang Ⅱ (1 μM; Figure 2F).

### 3.2 Trifolin attenuates L-type calcium channel-dependent constriction of isolated abdominal aortic rings

**FIGURE 3:**
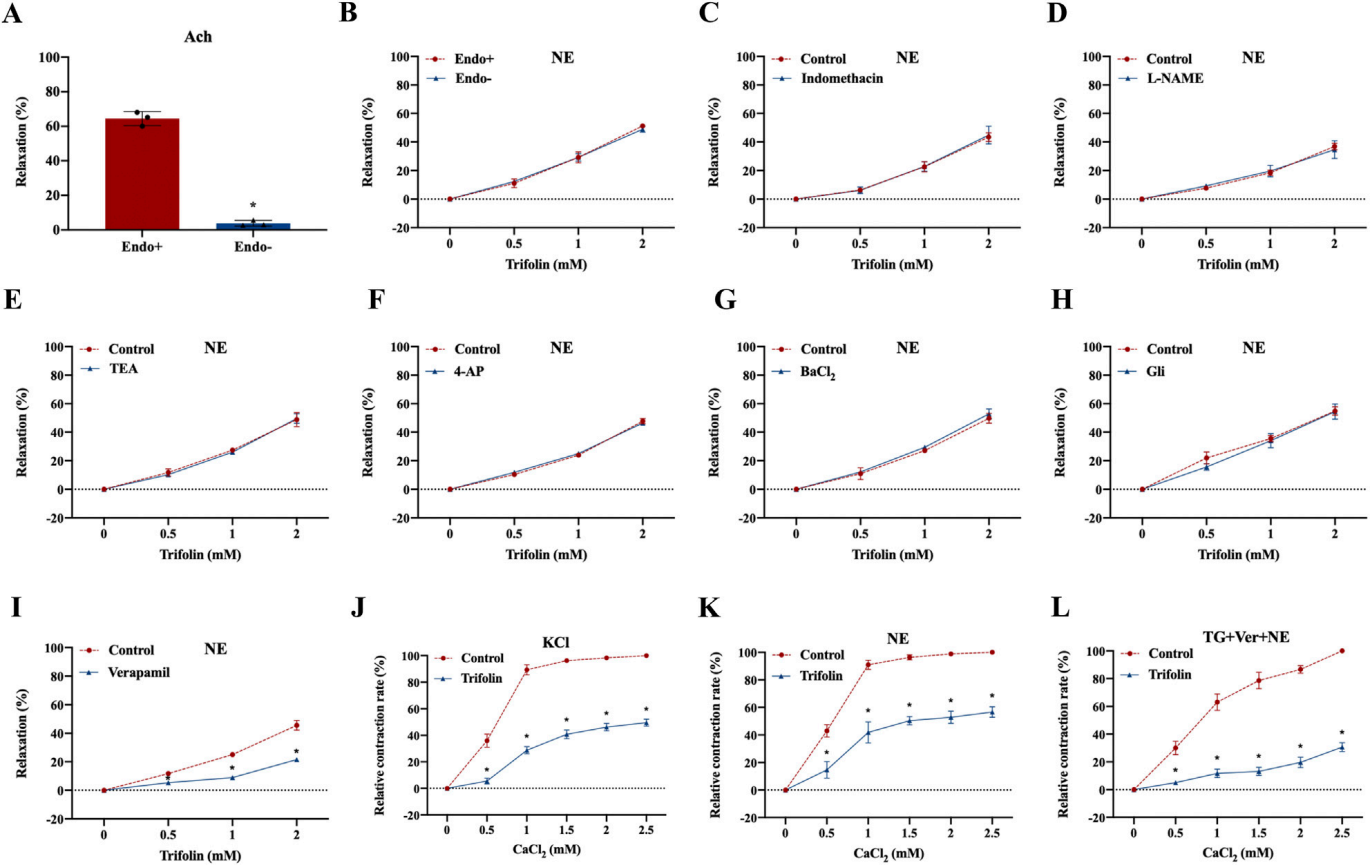
Trifolin attenuated calcium channel-dependent constriction of isolated abdominal aortic rings. Isolated abdominal aortic rings from Wistar rats were treated with various concentrations of trifolin (0.5, 1, or 2 mM). **(A)** Detection of the endothelium has been removed of aortic rings relaxed with acetylcholine (Ach, 10 μМ). **(B)** Precontraction of the endothelium-intact (Endo+) and endothelium-denuded (Endo-) aortic rings from Wistar rats with NE (1 μМ), followed by detection of the vasorelaxation of aortic rings treated with various concentrations of trifolin (0.5, 1, or 2 mM). **(C–D)** Endothelium-intact (Endo+) aortic rings from Wistar rats preincubated with endothelial inhibitors indomethacin (Indo, 10 μM) or L-NAME (0.1 mM) for 20 min, followed by precontracted with NE (1 μМ), and subsequently to detect the vasorelaxation of aortic rings treated with various concentrations of trifolin (0.5, 1, or 2 mM). Endothelium-denuded aortic rings preincubated with the K^+^ channel blockers, **(E)** TEA (0.1 mM), **(F)** 4-AP (0.1 mM), **(G)** BaCl_2_ (0.1 mM), or **(H)** Gli (0.01 mM) for 20 min, followed by NE (1 μМ) stimulation; after the plateau was attained, various concentrations of trifolin (0.5, 1, or 2 mM) are added, and the vasorelaxation of aortic rings is detected. **(I)** The endothelium-denuded aortic rings are preincubated with a L-type calcium channel inhibitor, verapamil (10 μМ), and precontracted with NE (1 μМ), followed by trifolin (0.5, 1, or 2 mM) treatment and detection of the vasorelaxation of the aortic rings. After incubation with EGTA, endothelium-denuded aortic rings are rinsed in Ca^2+^-free and **(J)** KCl, **(K)** NE or **(L)** thapsigargin (TG, 4 μМ) PSS solution, followed by CaCl_2_ (0.5, 1, 1.5, 2, or 2.5 mM) stimulation with or without trifolin treatment. Data were shown as the mean ± SD. *p < 0.05 vs. Control group.

To determine whether trifolin vasodilation is endothelium-dependent, we removed the endothelium of the aortic rings, leading to relaxation rate decrease, which was then treated with acetylcholine chloride (10 μМ) (Figure 3A). Moreover, NE stimulation increased the tension value on the endothelium-intact and endothelium-denuded aortic rings, but trifolin treatment significantly relaxed both rings (Figure 3B). As shown in Figures 3C,D, trifolin treatment yielded no different effect on the vasorelaxation of endothelium-denuded aortic rings after preincubation with the inhibitors of endothelial channel inhibitors [Indo (10 μM) or L-NAME (0.1 mM)]. Given that potassium and calcium channels both play significant roles as ion channels in VSMCs, we evaluated the vasorelaxant effect of trifolin treatment on the abdominal endothelium-denuded aortic rings after pretreatment with the blockers of K^+^ channels [TEA (0.1 mM), 4-AP (0.1 mM), BaCl_2_ (0.1 mM), or Gli (0.01 mM)]. We found that K^+^ channel blockers showed no effect on trifolin-induced vasorelaxation of the endothelium-denuded aortic rings precontracted with NE (Figures 3E–H). Conversely, pretreatment with the L-type calcium channel blocker verapamil (10 μМ) significantly decreased vasorelaxation enhancement following trifolin treatment on the endothelium-denuded aortic rings (Figure 3I). Furthermore, the assessment of the effect of trifolin on the constriction of isolated endothelium-denuded aortic rings indicated that trifolin treatment clearly alleviated CaCl_2_-induced constriction on such rings in the Ca^2+^-free solution containing KCl, NE, or TG (Figures 3J–L).

### 3.3 Trifolin attenuates aortic wall thickness on abdominal aorta in Ang Ⅱ-infused hypertensive mice

**FIGURE 4:**
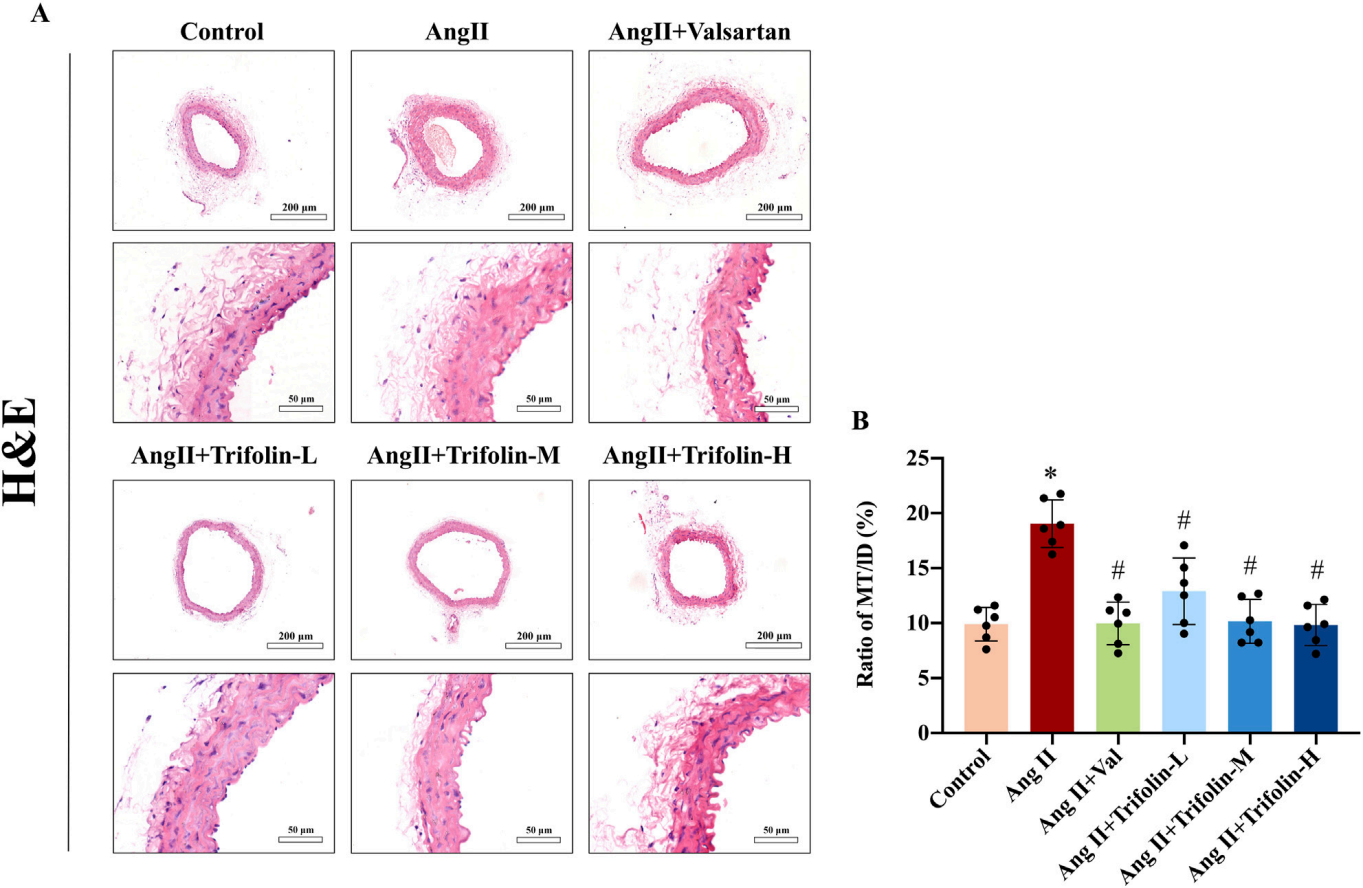
Trifolin alleviated aortic wall thickness on abdominal aorta in Ang Ⅱ-infused hypertensive mice. The effects of trifolin treatment on pathological changes in abdominal aortas of Ang Ⅱ-infused mice were determined by Hematoxylin-eosin (H&E) staining. **(A)** Representative H&E staining images of the abdominal aorta of the mice from each group were captured using a microscope at a magnification of ×400. **(B)** MT/LD was measured. Data were presented as mean ± SD; *p < 0.05 vs. Control group, #*p < 0.05* vs. Ang II group.

The effect of trifolin on morphological changes in Ang Ⅱ-infused hypertensive mice was determined using H&E staining. As shown in Figure 4, Ang Ⅱ infusion significantly increased the ratio of MT/LD compared with the control group, indicating that Ang Ⅱ infusion significantly thickened the abdominal aortic wall. After treatment with trifolin or alsartan, the ratio of MT/LD decreased significantly compared with the Ang Ⅱ group, suggesting that trifolin treatment could reduce the thickness of the abdominal aortic wall.

### 3.4 Trifolin inhibits the expression of SOC channel proteins on abdominal aorta in Ang Ⅱ-infused hypertensive mice

**FIGURE 5:**
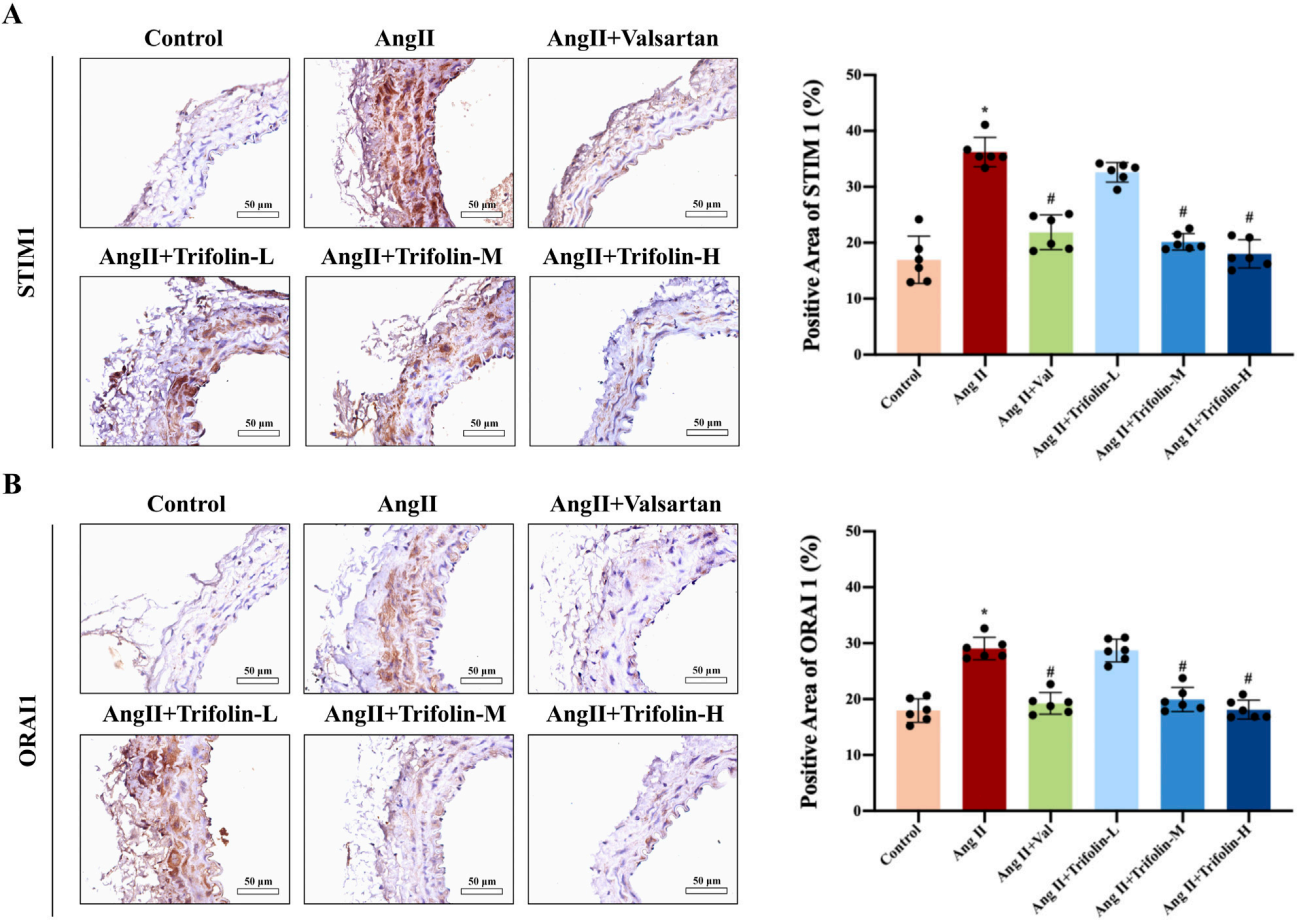
Trifolin attenuated the increased expression of STIM1 and ORAI1 in Ang Ⅱ-infused hypertensive mice. **(A,B)** The protein expression of STIM1 and ORAI1 from each group abdominal aorta was detected using immunohistochemistry. Representative images at ×400 magnification are shown. Data were presented as mean ± SD; *p < 0.05 vs. Control group, #*p < 0.05* vs. Ang Ⅱ group.

IHC was applied to determine the effect of trifolin on the expression of SOC channel proteins in Ang Ⅱ-infused hypertensive mice. The IHC analysis revealed that Ang Ⅱ infusion significantly upregulated the expression of STIM1 (Figure 5A) and ORAI1 (Figure 5B) in the abdominal aorta compared with controls. However, trifolin or valsartan treatment alleviated this effect.

### 3.5 Trifolin suppresses the activation of calcium signaling pathway on abdominal aorta in Ang Ⅱ-infused hypertensive mice

**FIGURE 6:**
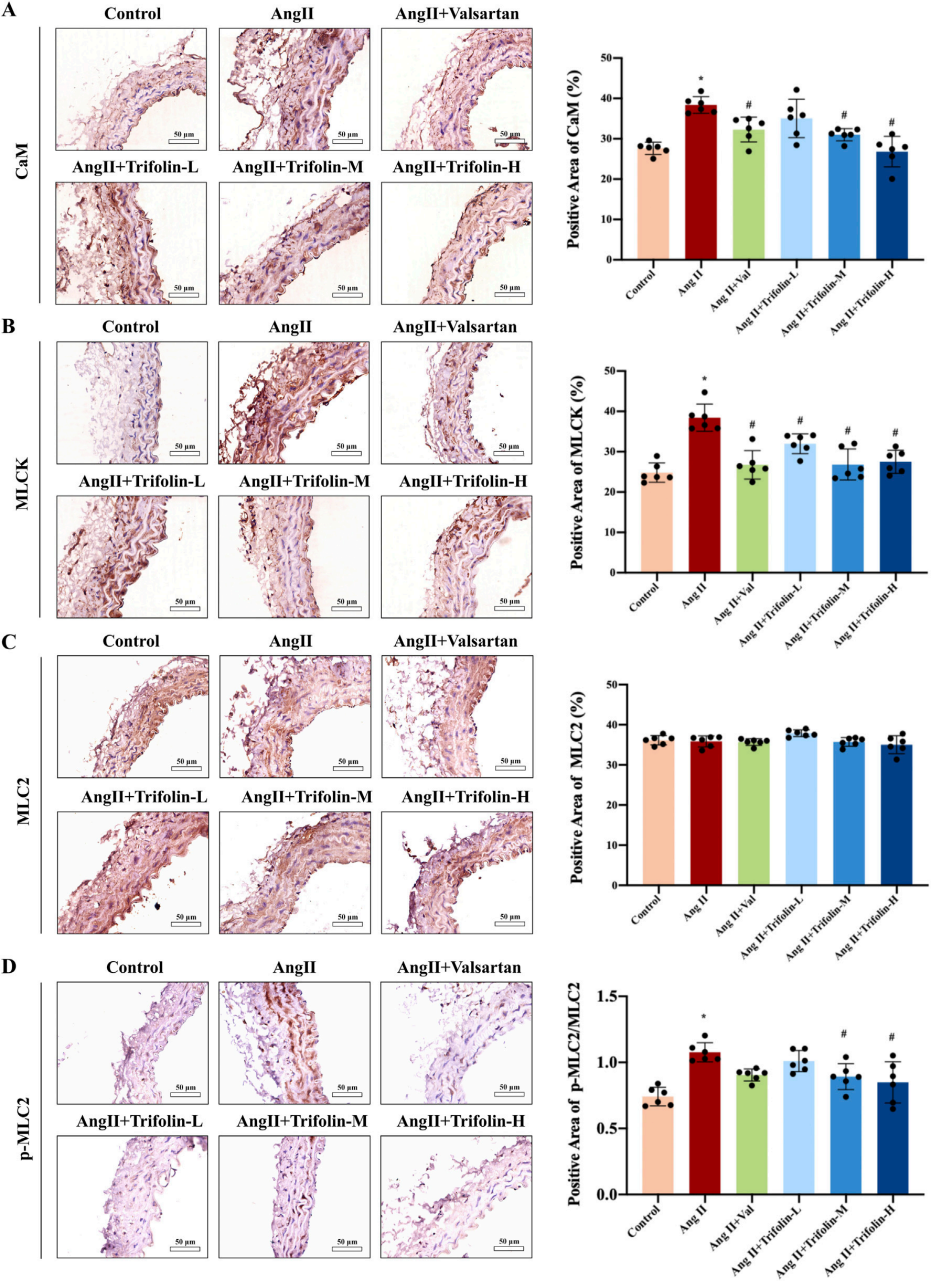
Trifolin suppressed the activation of CaM/MLCK/p-MLC2 pathway in Ang Ⅱ-infused hypertensive mice. **(A–D)** The protein expression of CaM, MLCK, MLC2, and p-MLC2 in abdominal aortas from each group was detected using immunohistochemistry. Representative images at ×400 magnification are shown. Scale bar = 50 μm. Data were presented as mean ± SD; *p < 0.05 vs. Control group, #*p < 0.05* vs. Ang Ⅱ group.

The regulatory effect of trifolin treatment on calcium pathway activation in Ang Ⅱ-infused hypertensive mice was evaluated using IHC. The IHC analysis showed that Ang Ⅱ infusion significantly increased the expression of the calcium signaling pathway proteins CaM (Figure 6A) and MLCK (Figure 6B) and the phosphorylation of MLC2 (Figures 6C,D) in the abdominal aortas as compared with controls. These effects were partially reversed after trifolin or valsartan treatment.

### 3.6 Trifolin reduces the stimulus-induced intracellular calcium increase in A7R5 cells

**FIGURE 7:**
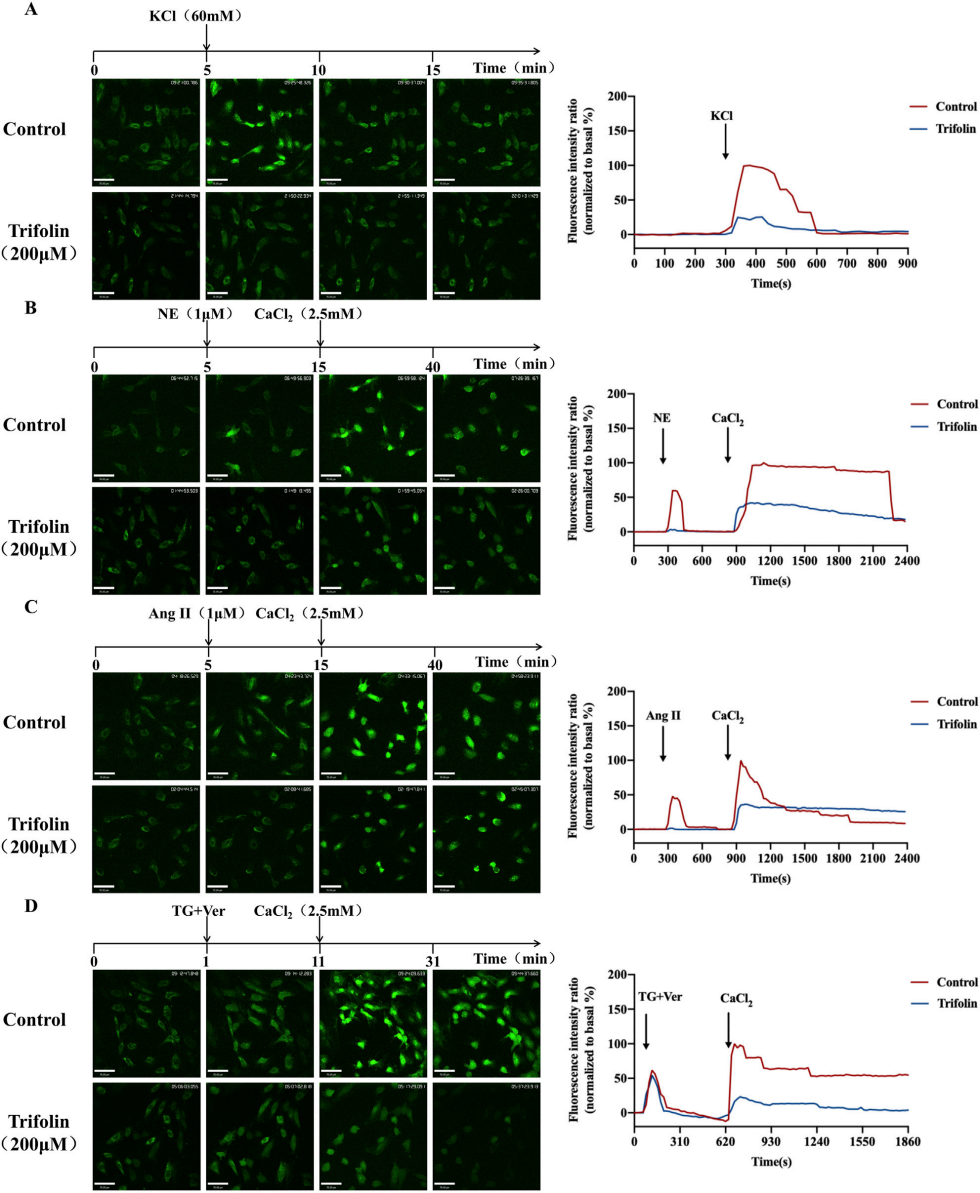
Trifolin reduced intracellular calcium increase in A7R5 cells. A7R5 cells were pretreated with 200 μМ, followed by KCl (60 mM), NE (1 μМ), Ang Ⅱ (1 μМ), and TG (4 μМ) stimulation. The intracellular calcium concentration in A7R5 cells was measured using a laser confocal focusing assay after fluo-4/AM staining. Images were taken with a confocal fluorescence microscope at a magnification of ×200. Real-time [Ca^2+^]i was monitored every 20 s, and the curve of the intracellular calcium concentration is drawn. **(A)** Trifolin inhibits KCl-induced calcium entry in A7R5 cells. A7R5 cells were placed in a calcium-free solution; then, after **(B)** NE, **(C)** Ang Ⅱ, or **(D)** TG stimulation, CaCl_2_ (2.5 mM) was added, and real-time [Ca^2+^]i was recorded during this process. Data were shown as the mean.

To further investigate the impact of trifolin on intracellular calcium release and extracellular calcium influx, we assessed the changes in intracellular calcium concentration in A7R5 cells by using a laser confocal focusing assay. Initially, the mean fluorescence intensity of the A7R5 cells in the resting state was comparable between the control and trifolin-treated groups. Then, KCl, NE, and Ang Ⅱ stimulations increased it, which was reduced by trifolin pretreatment (Figures 7A–C). After thapsigargin treatment, the mean fluorescence intensities did not significantly differ between the control and trifolin-treated groups (Figure 7D). Moreover, CaCl_2_ addition significantly increased the mean fluorescence intensity, which was inhibited by the trifolin pretreatment (Figures 7B–D).

### 3.7 Trifolin inhibits the expression of SOC channel proteins and calcium signaling pathway in Ang Ⅱ-stimulated A7R5 cells

**FIGURE 8:**
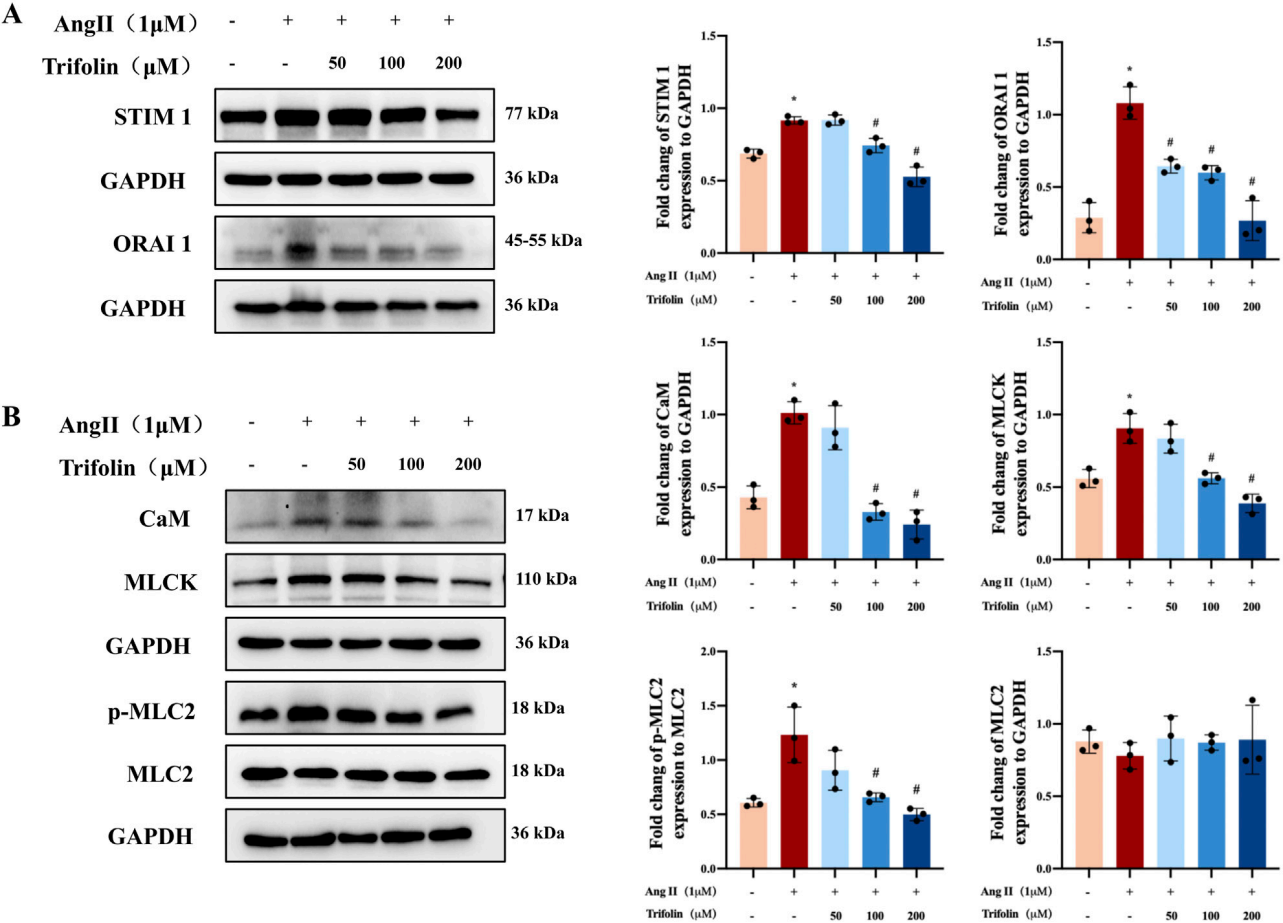
Trifolin attenuated the increased expression of SOC channel and calcium signaling-related proteins in A7R5 cells. **(A)** The expression of STIM1 and ORAI1 protein in Ang Ⅱ-stimulated A7R5 Cells after trifolin treatment was determined by western blot analysis, and GAPDH was used as the internal control. **(B)** The expression of CaM, MLCK, MLC2, and p-MLC2 proteins in Ang Ⅱ-stimulated A7R5 cells after trifolin treatment was determined by western blot analysis, and GAPDH was used as the internal control. Data were presented as mean ± SD; *p < 0.05 vs. the Control group, #*p < 0.05* vs. the Ang Ⅱ group.

To verify the regulatory effect of trifolin treatment on the expression of the SOC channel proteins and calcium signaling pathway proteins, we conducted western blotting, which revealed that Ang Ⅱ stimulation significantly upregulated the STIM1, ORAI1, CaM, and MLCK protein expression and MLC2 phosphorylation in A7R5 cells compared with the controls. However, they were downregulated after trifolin treatment (Figures 8A,B).

## 4 Discussion

Hypertension remains a major contributor to cardiovascular diseases, characterized by high morbidity and mortality worldwide (Labarthe and Dunbar, 2012; Pedrinelli et al., 2012). Despite existing treatments, there is an urgent need for innovative and effective therapeutic approaches with minimal risks are urgently needed (Scholze et al., 2006). Qingda Granules (QDG), a traditional Chinese medicine formula, reportedly lowers blood pressure effectively in hypertensive models (Yu et al., 2020; Cheng et al., 2021; Long et al., 2021). Among its bioactive components, trifolin, a flavonoid derived from QDG, has shown antihypertensive potential. In this study, after evaluating and ensuring the success of the hypertensive model, and confirming that trifolin exhibits favorable safety characteristics with undetectable drug toxicity of trifolin (Supplementary Figure S1A,B), we further investigated the underlying mechanisms of trifolin. We found that trifolin treatment inhibits vasoconstriction and calcium-related signaling pathway protein expression, providing mechanistic insights into its vascular effects.

Calcium signaling plays a pivotal role in regulating VSMC contraction, a critical determinant of maintaining blood pressure homeostasis (Allen and Walsh, 1994; Ikebe, 2008). An increase in intracellular calcium concentrations is a key trigger for VSMC contraction, primarily mediated by VDC, ROC, and SOC (Gees et al., 2010; Albert, 2011; Martinsen et al., 2014; Guzik et al., 2017). Our study demonstrated that trifolin significantly inhibited both calcium influx and release in VSMCs, leading to reduced vascular constriction. In vascular tension experiments, trifolin significantly attenuated the vasoconstriction induced by KCl, NE, and Ang Ⅱ, which are known inducers of calcium-dependent contraction in VSMCs. Therefore, trifolin may target both the extracellular calcium entry and intracellular calcium release pathways. Notably, the vasorelaxant effects of trifolin were independent of endothelial function, considering that the inhibitors of nitric oxide synthesis and cyclooxygenase did not affect its activity. These findings highlight the direct action of trifolin on VSMCs, particularly by inhibiting the calcium channels. Interestingly, the vasodilatory effect of trifolin was not affected by the T-type calcium channel blocker flunarizine dihydrochloride (Supplementary Figure S2A), but was partially reversed by the L-type calcium channel blocker verapamil and the store-operated calcium entry (SOCE) blocker YM-58483 (Supplementary Figure S2B), indicating that trifolin mainly interacts with or regulates the L-type calcium channels and SOCE. Further analysis revealed that trifolin inhibited calcium influx mediated by VDC, ROC, and SOC and reduced calcium release from intracellular stores induced by NE and Ang Ⅱ. Notably, in our study, there was no significant difference in cytoplasmic Ca^2+^ fluorescence intensity between the control group and the trifolin-treated group following TG treatment. This is likely because inhibition of SERCA by TG prevents endoplasmic reticulum Ca^2+^ reuptake, resulting in a transient increase in cytosolic calcium. As is well known, TG is a specific inhibitor of sarco/endoplasmic reticulum Ca^2+^-ATPase (SERCA), which actively pumps cytosolic Ca^2+^ back into the endoplasmic reticulum to maintain calcium store homeostasis (Sehgal et al., 2017). TG irreversibly binds to SERCA and inhibits its function, leading to the depletion of intracellular calcium stores due to impaired calcium recycling. Based on this theoretical foundation, we speculate that trifolin does not act through SERCA, which may explain the absence of a significant difference in fluorescence values between the groups. The above experiments have demonstrated that the dual regulation effect of trifolin, including the regulation of the extracellular calcium entry and intracellular calcium release, provides a comprehensive mechanism for the vasorelaxant and antihypertensive effects of trifolin.

SOC is crucial for maintaining calcium homeostasis in VSMCs. SOC activation, which is driven by the depletion of intracellular calcium stores, facilitates the calcium influx necessary for sustained vascular contraction (Tiruppathi et al., 2002; Dietrich et al., 2006). STIM1 and ORAI1 are key regulators of SOC activity. Located on the endoplasmic reticulum membrane, STIM1 senses calcium depletion and activates ORAI1 channels on the plasma membrane to replenish calcium stores (Stathopulos et al., 2006; Liou et al., 2007; Várnai et al., 2009). In our study, trifolin significantly downregulated STIM1 and ORAI1 expression in Ang Ⅱ-stimulated VSMCs, suggesting that trifolin can inhibit SOC-mediated calcium influx, contributing to its vascular effects. This observation aligns with trifolin’s ability to reduce thapsigargin-induced calcium release, further supporting its role in regulating SOC activity.

These channels facilitate calcium influx, activating downstream signaling pathways involving CaM and MLCK, leading to MLC2 phosphorylation and subsequently, vascular constriction (Means et al., 1991; Levinson et al., 2004; Martinsen et al., 2014; Li et al., 2015; Liu et al., 2017; Wan Omar et al., 2019; Fang et al., 2023). Our results revealed that Ang Ⅱ stimulation significantly upregulated CaM and MLCK expression and MLC2 phosphorylation, but trifolin treatment inhibited these effects, as confirmed by IHC and western blotting. By disrupting the CaM/MLCK/p-MLC2 pathway, trifolin interferes with the signals essential for VSMC contraction, effectively reducing vascular tension. These findings are consistent with prior studies that emphasized the critical role of this pathway in vascular responses to hypertensive stimuli.

Although our findings provide significant insights into the mechanisms underlying trifolin’s effects on calcium signaling and vascular function, several questions remain unanswered. One important question is whether trifolin directly interacts with calcium channels or modulates their activity via upstream regulators. Although our results suggest that trifolin reduces the expression of SOC-related proteins such as STIM1 and ORAI1, it is still not clear whether trifolin acts by directly inhibiting the interaction between STIM1 and ORAI1 or regulating the activity of MLCK. To elucidate these interactions, we need to conduct additional studies using specific inhibitors, genetic knockdown, and advanced techniques, such as co-immunoprecipitation and proteomics.

## 5 Conclusion

Our study provides compelling evidence that trifolin exerts its antihypertensive effects by regulating calcium signaling and inhibiting key constriction-related pathways, such as the CaM/MLCK/p-MLC2 pathway, in VSMCs. By targeting both extracellular calcium entry and intracellular calcium release, trifolin effectively reduces VSMC contraction. Therefore, trifolin can potentially be an innovative therapeutic agent for hypertension treatment, offering a unique mechanism of action. Future research should further explore its full therapeutic potential, including its effects on other signaling pathways, anti-inflammatory properties, and clinical efficacy in patients with hypertension.

## Data Availability

The datasets presented in this study can be found in online repositories. The names of the repository/repositories and accession number(s) can be found in the article/Supplementary Material.
