# Supplementary material for: Trifolin inhibits the calcium-driven contraction pathway in vascular smooth muscle
Source: Front Pharmacol. 2025 May 30;16:1573483. doi: 10.3389/fphar.2025.1573483 (PMC12162279; doi:10.3389/fphar.2025.1573483)
Supplement: Supplementary file 1 [file DataSheet1.pdf]

1 Result

1.1 Different concentrations of trifolin have no significant toxic side effects on Ang Ⅱ-infused hypertensive mice.

H&E staining of heart tissues from all groups showed no structural abnormalities or signs of inflammatory cell infiltration (supplementary Figure 1A). Moreover, serum levels of ALT, AST, BUN, and CRE showed no significant alterations in trifolin-treated groups compared to controls (supplementary Figure 1B).

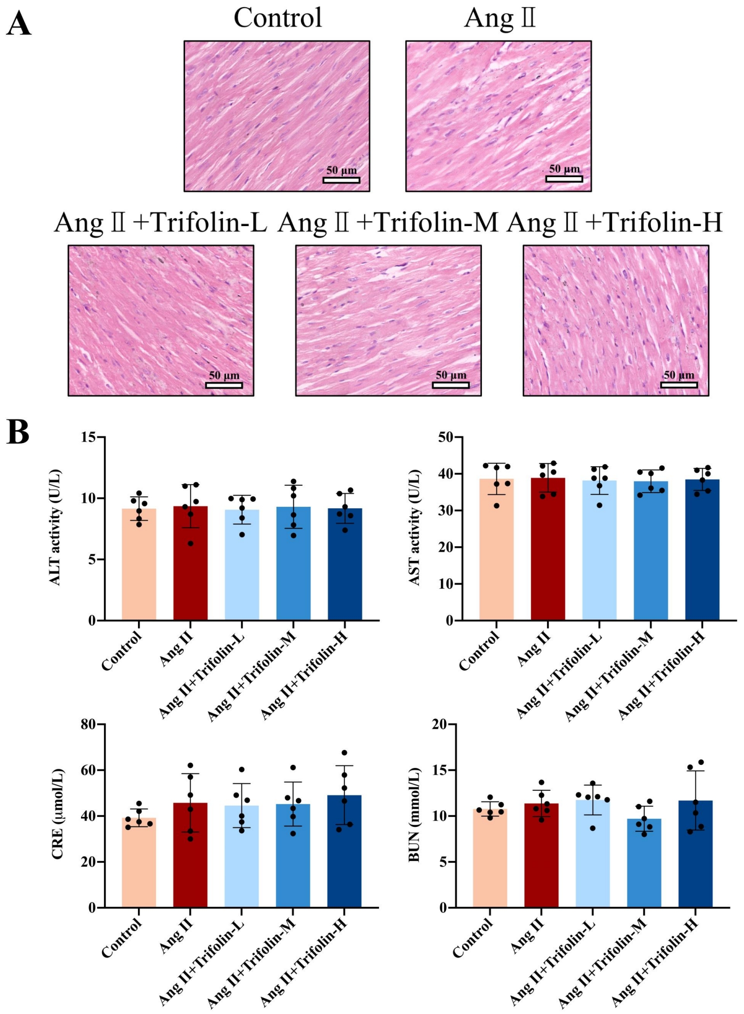

Figure 1. Safety detection of mice intervened with different concentrations of trifolin.

(A) Representative images of the left ventricles of each group after H&E staining. **(B)** The activity values of alanine aminotransferase (ALT) and aspartate aminotransferase (AST), which are liver function indicators in mouse serum, and the contents of blood urea nitrogen (BUN) and creatinine (CRE), which are kidney function indicators, were detected using kits. Data were presented as mean ± SD.

1.2 Trifolin Attenuates Store-operated Calcium Channel-dependent Constriction of Isolated Abdominal Aortic Rings

We evaluated the vasorelaxant effect of trifolin treatment on the abdominal endothelium-denuded aortic rings after pretreatment with the blocker of T-type calcium channel (flunarizine dihydrochloride [10 μM]). We found that -type calcium channel blocker showed no effect on trifolin-induced vasorelaxation of the endothelium-denuded aortic rings precontracted with NE (supplementary Figure 2A). Notably, pretreatment with the SOCE blocker YM-58483 (1 μM) significantly decreased vasorelaxation enhancement following trifolin treatment on the endothelium-denuded aortic rings (supplementary Figure 2B).

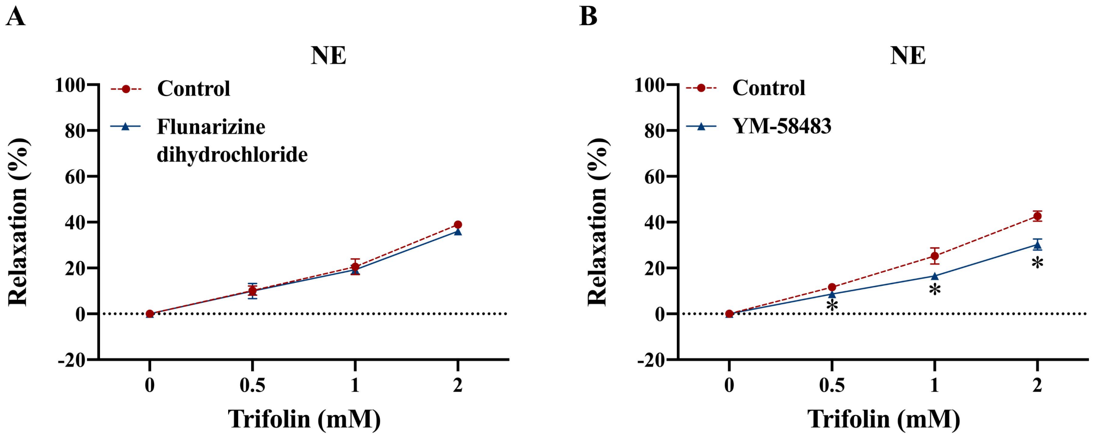

Figure 2. Detection of the dependence of trifolin on calcium ion channels.

Isolated abdominal aortic rings from Wistar rats were treated with various concentrations of trifolin (0.5, 1, or 2 mM). The endothelium-denuded aortic rings are preincubated with **(A)** T-type calcium channel blocker flunarizine dihydrochloride (10 μM) or **(B)** SOCE blocker YM-58483 (1 μM), and precontracted with NE (1 μM), followed by trifolin (0.5, 1, or 2 mM) treatment and detection of the vasorelaxation of the aortic rings. Data were presented as mean ± SD; *$p < 0.05$ vs. Control group.
